# Immunostimulatory Effects of Gamisoyosan on Macrophages via TLR4-Mediated Signaling Pathways

**DOI:** 10.3390/nu16193266

**Published:** 2024-09-27

**Authors:** Yun Hee Jeong, Wei Li, Hye Jin Yang, Jang-Gi Choi, You-Chang Oh

**Affiliations:** Korean Medicine (KM)-Application Center, Korea Institute of Oriental Medicine, 70, Cheomdanro, Dong-gu, Daegu 41062, Republic of Korea; runxi0333@kiom.re.kr (Y.H.J.); liwei1986@kiom.re.kr (W.L.); hjyang@kiom.re.kr (H.J.Y.); jang-gichoi@kiom.re.kr (J.-G.C.)

**Keywords:** immunoregulation, macrophage, protein kinase B, mitogen-activated protein kinase, nuclear factor-κB, Toll-like receptor 4, gamisoyosan

## Abstract

Background: This study aimed to analyze the immunostimulatory activity of gamisoyosan (GSS) on the activation of macrophages in RAW 264.7 cells and its underlying mechanisms. Methods: The effects of GSS on the secretion of nitric oxide (NO), immunomodulatory mediators, cytokines and mRNAs, and related proteins were assessed using the Griess assay, Western blotting, quantitative polymerase chain reaction, enzyme-linked immunosorbent assay, and H_2_DCFDA, respectively. The level of phagocytosis was determined by the neutral red method while the immune function of GSS was determined using adhesion and wound-healing assays. Results: GSS-treated macrophages significantly increased the production of NO, immunomodulatory enzymes, cytokines, and intracellular reactive oxygen species without causing cytotoxicity. GSS effectively improved macrophage immune function by increasing their phagocytic level, adhesion function, and migration activity. Mechanistic studies via Western blotting revealed that GSS notably induced the activation of the Toll-like receptor (TLR) 4-mediated mitogen-activated protein kinase, nuclear factor-κB, and protein kinase B signaling pathways. Conclusions: Overall, our results indicated that GSS could activate macrophages through the secretion of immune-mediated transporters via TLR4-dependent signaling pathways. Thus, GSS has potential value as an immunity-enhancing agent.

## 1. Introduction

The immune system is critical in providing protection from infections, healing damage, and maintaining homeostasis [1]. An ideal immunostimulation can enhance the immune defense system against external pathogens by controlling the activation of immune cells [2]. Macrophages are important innate immune cells that participate in phagocytosis, recognition of foreign substances, production of antibodies, and killing of abnormal cells [3,4]. Furthermore, macrophages can initiate the adaptive immune response through presenting antigens and secreting cytokines [5]. Thus, macrophage activation is considered a promising target for enhancing the immune systems of individuals with weak or impaired immune systems.

Toll-like receptors (TLR) are central molecules in the innate immune system. When TLR signaling is activated, pathogen-associated molecular patterns (PAMPs) are recognized, resulting in an innate immune response, which is then stimulated to initiate an adaptive immune response [6,7]. TLR4 is the most important membrane receptor expressed on innate immune cells, including dendritic cells and macrophages. Ligand-binding to TLR4 activates the transcription factor nuclear factor (NF)-κB and mitogen-activated protein kinase (MAPK) signaling pathways through the myeloid differentiation factor (MyD)88-dependent pathway, which is a crucial regulator of the immune response. Activation of these pathways induces the production of immunomodulatory factors, such as cytokines, nitric oxide (NO), and synthesizing enzymes, including inducible nitric oxide synthase (iNOS) and cyclooxygenase (COX)-2 [8]. Additionally, TLR4 contributes to improving immunity by activating the protein kinase B (Akt) signaling pathway [9].

Gamisoyosan (GSS) contains 11 types of medicinal herbs, and according to a traditional Korean medicinal book, *Dongeuibogam*, is effective in relieving blood deficiency, irritable fever, cold sweat, flushing, phlegm, and cough [10]. GSS is commonly used to treat women with menopausal conditions and related symptoms in countries such as Korea, China, and Japan [11]. A recent study reported that GSS has a positive effect on ovulation and in vitro fertilization in mice stressed by electric shock [12]. Another study showed that GSS had an improving effect on premenstrual dysphoric disorder in women [13]. However, the effect of GSS on enhancing immunity through macrophage activation has not been previously studied. Therefore, we researched the immunostimulatory effect of GSS decoction and its mechanism in RAW 264.7 cells. Additionally, the constituents of GSS were confirmed through UHPLC-UV-MS/MS-based phytochemical analysis.

## 2. Materials and Methods

### 2.1. Materials and Reagents

Antibiotics, fetal bovine serum (FBS), and Dulbecco’s modified Eagle medium (DMEM) were acquired from Hyclone (Logan, UT, USA). Culture dishes, well plates, and all plasticware used for cell culture were obtained from SPL (Pocheon, Republic of Korea). A cell counting kit (CCK) was purchased from Dojindo Laboratories (Kumamoto, Japan). Phosphoric acid, N-1-napthylethylenediamine dihydrochloride, and sulfanilamide for the Griess reagent were obtained from Sigma-Aldrich (St. Louis, MO, USA). Enzyme-linked immunosorbent assay (ELISA) kits were acquired from Invitrogen (Waltham, MA, USA). An RNA extraction kit, DNA synthesizing kits, and SYBR green master mix were purchased from Bioneer (Daejeon, Republic of Korea) and iNtRON (Sungnam, Republic of Korea), respectively. H_2_DCFDA, neutral red, crystal violet, and bovine serum albumin (BSA) were acquired from Sigma-Aldrich. Antibodies to TLR4 (cat. no. 14358), MyD88 (cat. no. 4283), β-actin (cat. no. 4970), P-Akt (cat. no. 4060), Akt (cat. no. 9272), P-ERK (cat. no. 4377), ERK (cat. no. 9102), P-p38 (cat. no. 9211), P38 (cat. no. 9212), P-JNK (cat. no. 9251), JNK (cat. no. 9252), P-IκBα (cat. no. 2859), IκBα (cat. no. 4814), P-NF-κB p65 (cat. no. 3033), NF-κB p65 (cat. no. 8242), Lamin B1 (cat. no. 13435), iNOS (cat. no. 13120), and COX-2 (cat. no. 4842), as well as secondary antibodies, were obtained from Cell Signaling Technology (Boston, MA, USA).

### 2.2. Preparation of GSS

The 11 herbs that make up GSS (Table 1) were all purchased from Humanherb (Daegu, Republic of Korea). Prior to extraction, Dr. Wei Li of the Korea Institute of Oriental Medicine (KIOM, Daegu, Republic of Korea) verified all herbs constituting the GSS, and 11 herb samples were deposited in the herbal bank of KIOM with the identification number C24-17. The dried herbs (36.0 g) were extracted with 1 L of boiling water for 3 h. The decoction was filtered and freeze-dried, and the GSS powder was kept at −20 °C.

### 2.3. Macrophage Cell Culture and Drug Treatment

RAW 264.7 cells, a mouse macrophage cell line, were acquired from the Korean Cell Line Bank (Seoul, Republic of Korea). Cells were cultured in completed DMEM supplemented with 10% FBS, 100 U/mL penicillin, and 100 μg/mL streptomycin at 37 °C in 5% CO_2_. Macrophages were treated with several concentrations of GSS (10–200 μg/mL) or lipopolysaccharide (LPS) (10 ng/mL) for 30 min to 72 h.

### 2.4. CCK Assay for Cell Viability

The cytotoxicity of GSS to RAW 264.7 macrophages was determined using the CCK assay. RAW 264.7 cells were seeded at 5 × 10^4^ per well in 96-well culture plates for 18 h. The RAW264.7 cells were treated with different doses of GSS (10–200 μg/mL) or LPS (10 ng/mL). After 24–72 h of incubation, they were stained with 10 μL of CCK solution for an additional 1 h at 37 °C. The optical absorption at 450 nm was assessed using a microplate spectrophotometer (Molecular Devices, San Jose, CA, USA).

### 2.5. Nitrite Assay

The NO production in the culture supernatant was evaluated using the Griess reaction test. RAW 264.7 cells were seeded at a density of 5 × 10^4^ per well in 96-well plates. The cells were treated with different concentration of GSS or LPS for 24–72 h; LPS was used as the positive control. Griess reagent was mixed with a same volume of supernatant and incubated for five minutes at room temperature (RT). The optical density was then detected at 570 nm using a microplate spectrophotometer.

### 2.6. Western Blot Analysis

After treatment and incubation were completed, the cells were washed twice with PBS and lysed with a RIPA lysate buffer (Millipore, Bedford, MA, USA) containing a phosphatase and protease inhibitor (Roche, Basel, Switzerland). Extracted nuclear and cytoplasmic proteins were fractionated by nuclear extraction kit (Thermo Fisher Scientific, Waltham, MA, USA). The protein content was analyzed by a BCA kit (Thermo Fisher Scientific) according to the manufacturer’s instruction. Equivalent amounts of protein were used in SDS-PAGE electrophoresis at 60 V and electrotransferred onto PVDF membranes at 30 V overnight in an ice bath. Non-specific binding sites of membranes were blocked with 3% BSA at room temperature for 1 h. After blocking, the membranes were further incubated with each primary antibody overnight at 4 °C in a shaking incubator. The membranes were washed four times with TBS-T for 10 min and probed with horseradish peroxidase-conjugated secondary antibodies. The blots were exposed using a ChemiDoc imaging system (Bio-Rad, Hercules, CA, USA).

### 2.7. RNA Extraction, DNA Synthesis, and Quantitative Polymerase Chain Reaction (qPCR)

Total RNA extracts were prepared with an RNA extraction kit. Isolated RNA was reverse-transcribed into cDNA. The qPCR assay was performed using SYBR green master mix and synthetic primers following the standard protocol. qPCR was performed by a Real-time PCR System (Thermo Fisher Scientific). The level of β-actin mRNA was assessed as the normalization control; primer sequences are listed in Table 2.

### 2.8. Evaluation of Cytokine Secretion

The level of cytokines in the culture medium was calculated using ELISA kits. Cells (2.5 × 10^5^ per well) were seeded on 24-well culture plates and exposed to GSS or LPS for 24–72 h. Supernatants were then harvested by centrifugation, and the production of cytokines was determined using an ELISA reader at 450 nm absorbance.

### 2.9. Analysis of Intracellular Reactive Oxygen Species (ROS) Production

The production of ROS in macrophages was evaluated using H_2_DCFDA. Cells (5 × 10^4^ per well) were seeded in 96-well culture plates and exposed to GGS or LPS for 6 h. The culture media was gently discarded, and cells were stained using 15 μM of H_2_DCFDA at 37 °C for 30 min. The medium was removed, and stained cells were washed two times with PBS. ROS production was estimated by ELISA reader at 488 nm excitation and 525 nm emission. The fluorescence was observed by a fluorescence microscope (Eclipse Ti, Nikon, Tokyo, Japan).

### 2.10. Phagocytosis Assay

Macrophage phagocytosis activity was measured as described in a previous study [14]. Cells (1 × 10^4^ per well) were seeded on 96-well culture plates and treated with GSS or LPS for 24 h. The medium was discarded and washed gently with PBS three times, and 0.1% neutral red solution was stained per well and further incubated at 37 °C for 10 min. Following three further PBS washes, cell lysis buffer (ethanol: 1% glacial acetic acid = 1:1) was added per well and incubated for 2 h at RT. A microplate reader was used to determine the absorbance at 540 nm. The phagocytic index was quantified as previously described [15].

### 2.11. Adhesion Function

Cells were seeded at a density of 1 × 10^6^ per well on 6-well culture plates following GSS (10–200 μg/mL) or LPS treatment for 24 h. Subsequently, cells were collected by scraping, centrifuging, and reinoculating on 24-well culture plates at 2.5 × 10^5^ cells per well for 1 h. The media were discarded, and the cells were washed 3 times with PBS and then fixed with 0.5 mL of 4% paraformaldehyde for 15 min. Each well was then washed three times with PBS and added to a 0.2% crystal violet solution for 15 min. The stained cells were gently washed two times with PBS. Representative images were acquired using an inverted microscope.

### 2.12. Wound-Healing Assay

Cells (5 × 10^5^ per well) were seeded on 12-well culture plates and incubated for more than 24 h. When the confluency of the cells was ideal, wounds were generated using a 200 μL micropipette tip across the center of the well. Cells were then treated with GSS or LPS for 24 h. Scratch images were captured at 0 and 24 h by an inverted microscope, and wound areas were calculated using Image J software (Version 1.53e, National Institutes of Health, Bethesda, MD, USA).

### 2.13. UHPLC-UV-MS/MS Analysis

To identify phytochemicals in GSS, MS/MS analysis was conducted using an ultrahigh-performance liquid chromatography system coupled with a quadrupole-orbitrap mass spectrometer (UHPLC-UV-HRMS, Thermo Fisher Scientific) under previously established conditions [16]. The chromatographic separation of GSS components was achieved on an Acquity BEH C18 column (100 × 2.1 mm, 1.7 μm, Waters) maintained at 40 °C for 20 min. For optimal separation, the mobile phase comprised water with 0.1% (*v*/*v*) formic acid (A) and acetonitrile (B). MS-grade methanol, acetonitrile, water, and formic acid were procured from Thermo Fisher Scientific. Reference standards were obtained from Sigma-Aldrich, Targetmol (Wellesley Hills, MA, USA), and ChemFaces (Wuhan, China), all with a purity >97%. All data acquired in this study were processed and analyzed using Xcalibur v.4.2 and Tracefinder v.4.0 software (Thermo Fisher Scientific).

### 2.14. Statistical Analysis

The data were statistically analyzed using GraphPad Prism 8.0 software. After comparing each sample, numerical statistics were examined with one-way analysis of variance with Dunnett’s test. The results were acquired from three or more independent experiments. The significance of the data was measured at *p* values of * < 0.05, ** < 0.01, and † < 0.001 (vs. control).

## 3. Results

### 3.1. GSS Induces Secretion of NO and Expression of iNOS and COX-2 in Macrophages at Noncytotoxic Concentrations

**Figure 1:**
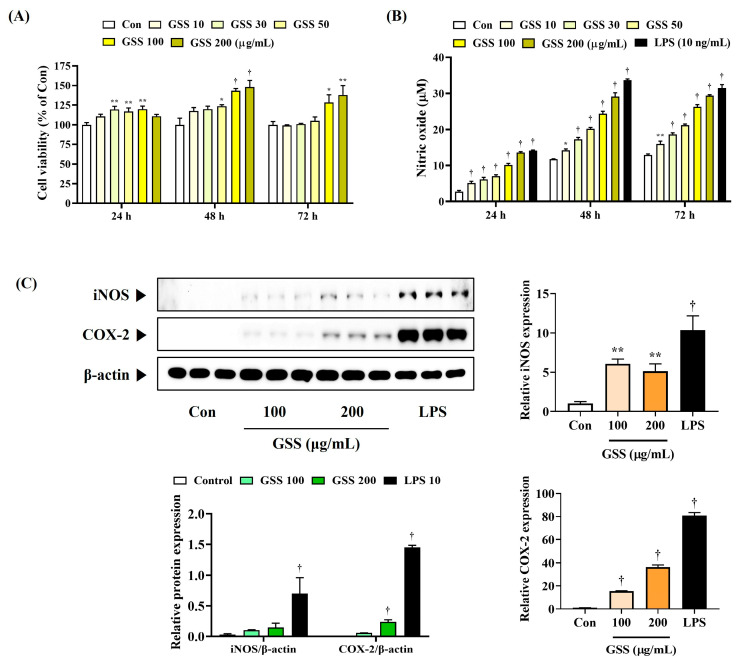
Effects of GSS on the (**A**) cell viability, (**B**) secretion of NO, and (**C**) level of iNOS and COX-2 in RAW 264.7 macrophages. RAW 264.7 cells were incubated with GSS or LPS for (**A**,**B**) 24, 48, or 72 h, or (**C**) 24 h. Data were acquired from three or more independent experiments. A *p* value less than 0.05 (vs. control) was considered statistically significant.

First, in the CCK assay performed to investigate the effect of GSS on macrophage cell viability, we confirmed that GSS did not show cytotoxicity up to a concentration of 200 μg/mL (Figure 1A). Figure 1A shows that no cytotoxicity was observed at any time after 24, 48, and 72 h of culture after GSS treatment.

Next, we measured the secretion of NO in RAW 264.7 macrophages to determine the effect of GSS on macrophage activation. Compared with the untreated control cells, GSS significantly and concentration-dependently induced NO secretion, which was more effective when cultured for 48 or 72 h than when cultured for 24 h (Figure 1B). We compared the efficacy of GSS using LPSs from Gram-negative bacteria, known as a TLR4 ligand, as a positive control.

Also, iNOS and COX-2 synthesize NO and PGE_2_, respectively, which play important roles in regulating immune responses in macrophages, and we therefore investigated the effects of GSS on the protein and mRNA levels of iNOS and COX-2. The iNOS and COX-2 protein levels were elevated in GSS-treated cells compared with those in the nontreated control (Figure 1C). Similarly, the respective mRNA levels were also significantly upregulated by GSS treatment.

### 3.2. GSS Effectively Induces Cytokine Secretion and Its mRNA Expression in Macrophages

Next, we analyzed the effects of GSS on the production of various cytokines associated with macrophage activation by ELISA and qPCR. In addition, we tested various incubation times to observe any changes in the immune response when macrophages were exposed to GSS for a long time.

**Figure 2:**
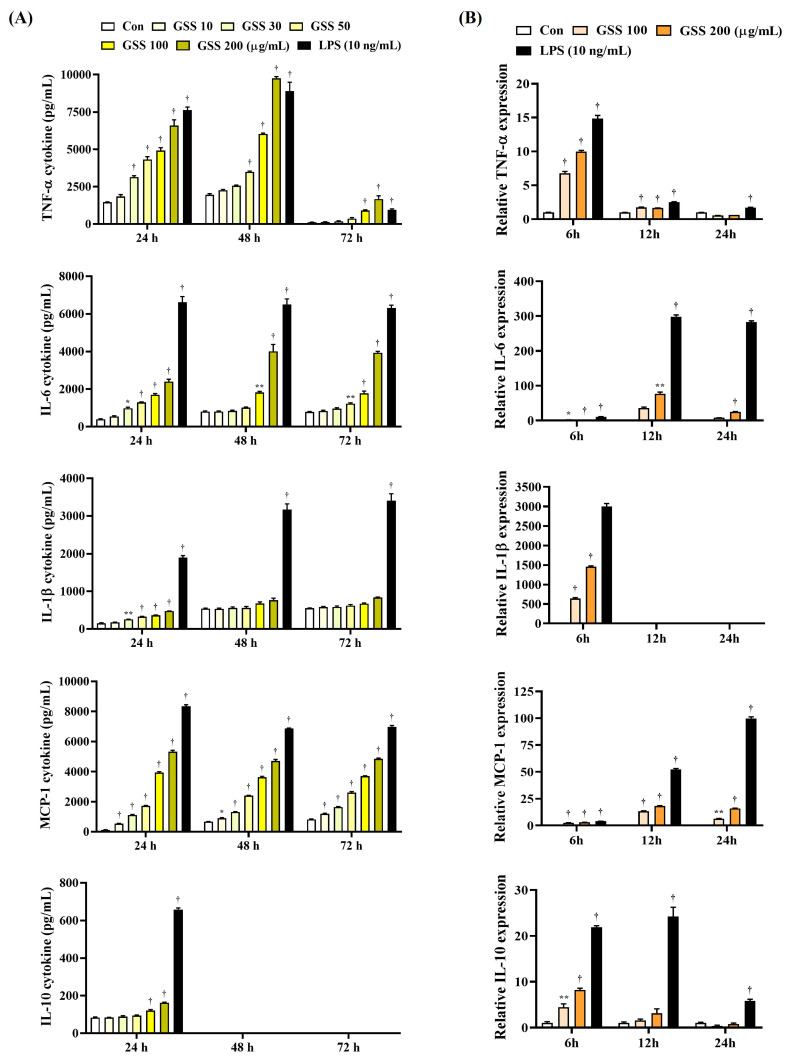
Effects of GSS on the (**A**) secretion of cytokines and the (**B**) level of cytokine mRNAs in macrophages. RAW 264.7 cells were incubated with GSS or LPS for (**A**) 24, 48, or 72 h, or (**B**) 6, 12, or 24 h. Data were acquired from three or more independent experiments. A *p* value less than 0.05 (vs. control) was considered statistically significant.

Compared with the untreated control cells, GSS significantly elevated the production of cytokines TNF-α, IL-6, IL-1β, MCP-1, and IL-10 in a concentration-dependent manner (Figure 2A). TNF-α cytokine was secreted most effectively when GSS was treated for 24 h compared with the untreated control cells, and no significant effect was observed when treated for 72 h. IL-6, IL-1β, and MCP-1 cytokines were similarly induced at all time points, but showed the highest significance at 24 h compared with the untreated control cells. The IL-10 cytokine was significantly induced at 24 h and was not detected at other time points. Therefore, in summary, the efficacy of GSS for cytokine induction was most effective when cultured for 24 h, and it was shown that a certain level of immune activation effect was maintained even for a longer time (Figure 2A).

The results of cytokine mRNA level analysis showed a similar trend to the induction efficacy of GSS for cytokine secretion, but there were differences depending on the culture time period. Compared to the untreated control cells, the expression of TNF-α, IL-1β, and IL-10 mRNA increased most effectively when cultured for 6 h with GSS treatment. In addition, the expression of IL-6 and MCP-1 mRNA increased most effectively when cultured for 12 h (Figure 2B). Therefore, the induction effect of GSS on cytokine mRNA expression is thought to reach its peak at a relatively early time.

### 3.3. GSS Strongly Increases the Production of Intracellular ROS in Macrophages

**Figure 3:**
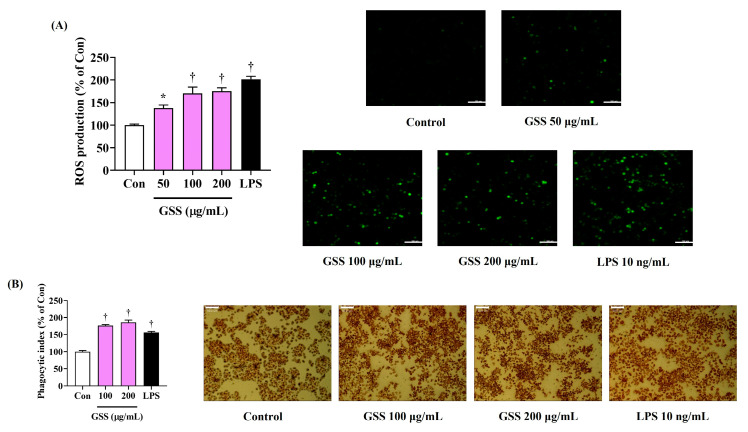
Effects of GSS on (**A**) the production of intracellular ROS and (**B**) the phagocytic activity in RAW 264.7 macrophages. Cells were incubated with GSS or LPS for (**A**) 6 h or (**B**) 24 h. Scale bar = (**A**) 100 μm or (**B**) 50 μm. Data were acquired from three or more independent experiments. A *p* value less than 0.05 (vs. control) was considered statistically significant.

We then used H_2_DCFDA, a fluorescent ROS indicator, to investigate the effects of GSS on the production of ROS in RAW 264.7 macrophages. LPS treatment notably increased the intracellular ROS accumulation, whereas untreated cells had a weak fluorescence intensity and low ROS content (Figure 3A). Additionally, GSS treatment significantly increased ROS production in a concentration-dependent manner, and, in particular, 200 μg/mL of GSS dramatically enhanced ROS expression to a level similar to that seen in the LPS treatment (Figure 3A).

### 3.4. Enhancing Effects of GSS on Macrophage Phagocytosis

The immunostimulatory activities of GSS on the effects of RAW264.7 cell phagocytosis were primarily determined using a neutral red uptake assay. GSS-treated cells exhibited an increase in phagocytic activity compared with that in the nontreated control, with a significant and slight concentration dependence observed (Figure 3B).

### 3.5. Enhancing Effect of GSS on Macrophage Immune Function

**Figure 4:**
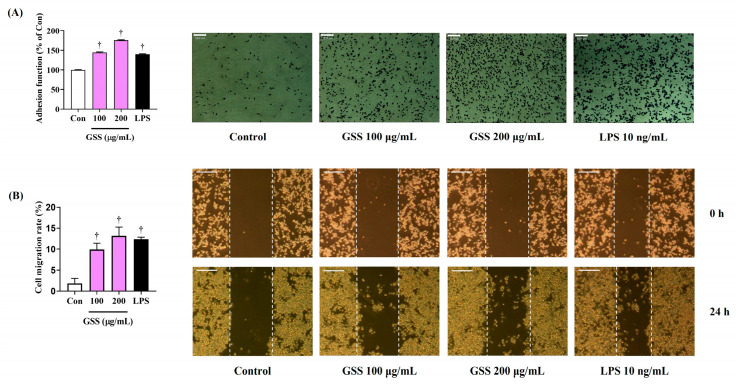
Effects of GSS on the (**A**) adhesion function and (**B**) migration function of RAW 264.7 macrophages. Cells were incubated with GSS or LPS for 24 h. Scale bar = (**A**) 50 μm or (**B**) 100 μm. Data were acquired from three or more independent experiments. A *p* value less than 0.05 (vs. control) was considered statistically significant.

To determine the impact of GSS on the immunomodulation of RAW 264.7 macrophages, we examined their adhesion function and migration activity. Crystal violet staining showed that GSS significantly improved the adhesion function of RAW 264.7 macrophages in a concentration-dependent manner compared with that of the nontreated control (Figure 4A). Additionally, the effects of GSS treatment on the migration activity of RAW 264.7 macrophages were investigated through a wound-healing assay, which demonstrated that GSS effectively elevated the proliferation and migration effect of RAW264.7 cells compared with those of the nontreated control (Figure 4B).

### 3.6. GSS Effectively Activates Akt, MAPK, and NF-κB Pathways by Activating TLR4

**Figure 5:**
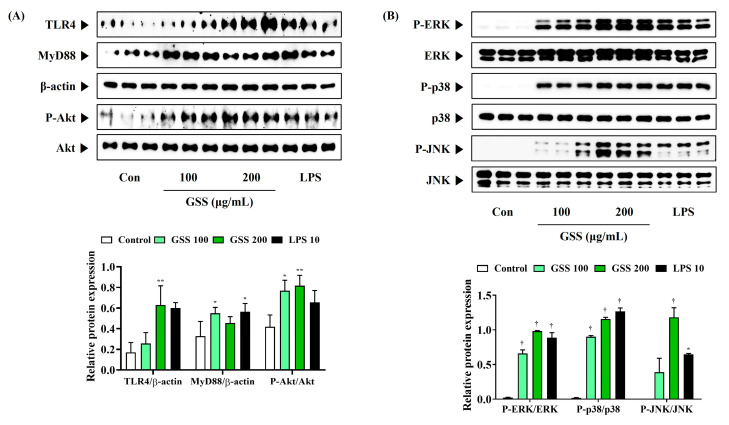
Effects of GSS on (**A**) the activation of TLR4/Akt and (**B**) the phosphorylation of MAPK in RAW 264.7 macrophages. Cells were incubated with GSS or LPS for 30 min. Histogram graphs show the production levels of protein relative to β-actin or total-form protein. Data were acquired from three independently obtained protein samples. A *p* value less than 0.05 (vs. control) was considered statistically significant.

To understand the molecular mechanisms underlying GSS-mediated macrophage activation in more depth, we evaluated whether GSS could activate the TLR4-mediated Akt, MAPK, and NF-κB signaling pathways. GSS increased the expression of TLR4 compared with that in control cells and enhanced MyD88 expression (Figure 5A). GSS also effectively induced Akt phosphorylation (Figure 5A). Similarly, ERK, p38, and JNK phosphorylation was strongly upregulated by GSS treatment in macrophages, indicating that the MAPK pathway was activated by GSS (Figure 5B).

**Figure 6:**
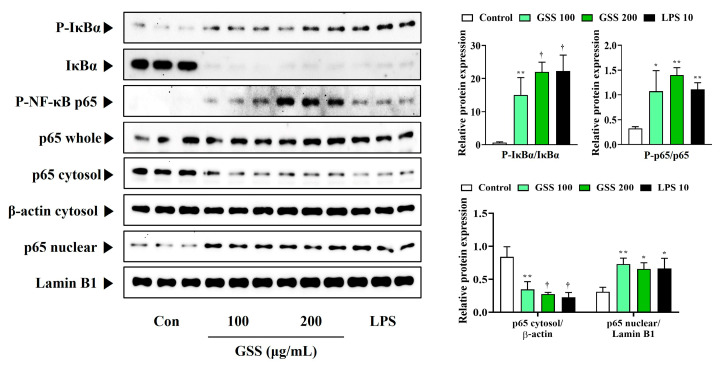
Effects of GSS on the activation of NF-κB in RAW 264.7 macrophages. Cells were incubated with GSS or LPS for 1 h. Histogram graphs show the production levels of protein relative to total-form protein, β-actin, or Lamin B1. Data were acquired from three independently obtained protein samples. A *p* value less than 0.05 (vs. control) was considered statistically significant.

Additionally, we found that GSS treatment significantly promoted the phosphorylation and degradation of IκBα, with significantly increased nuclear translocation and phosphorylation of p65 (Figure 6). Western blotting showed that GSS treatment could enhance the immune response of macrophages via the activation of TLR4-mediated Akt, MAPK, and NF-κB signaling pathways.

### 3.7. TAK-242 Inhibits the Immune Activation Effect of GSS

**Figure 7:**
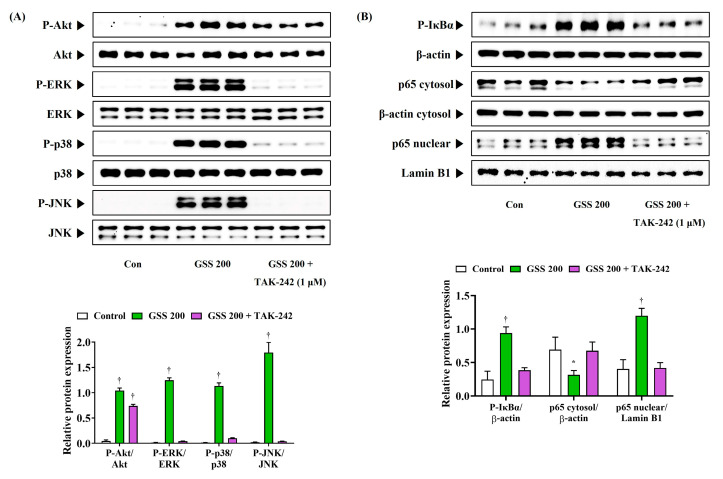
Effects of TAK-242 on the activation of (**A**) Akt/MAPK and (**B**) NF-κB in RAW 264.7 macrophages. Cells were incubated with GSS or TAK-242 for (**A**) 30 min or (**B**) 1 h. Histogram graphs show the expression levels of protein relative to total-form protein, β-actin, or Lamin B1. Data were acquired from three independently obtained protein samples. A *p* value less than 0.05 (vs. control) was considered statistically significant.

To determine whether GSS affects the activation of macrophages through the regulation of TLR4-mediated pathways, we analyzed how the activation of the Akt, MAPK, and NF-κB signaling pathways were changed using the TLR4 inhibitor TAK-242. As shown in Figure 7A, GSS treatment alone strongly induced the phosphorylation of Akt, ERK, p38, and JNK, which clearly activated the Akt and MAPK pathways. However, the activation of this mechanism was significantly inhibited when GSS and TAK-242 were simultaneously treated (Figure 7A). In addition, the phosphorylation of IκBα and the nuclear translocation of p65, which were rapidly increased by GSS treatment alone, were strongly inhibited by the combined treatment with TAK-242 (Figure 7B). This suggests that the inhibition of TLR4 activation by TAK-242 treatment also strongly inhibited the activation of its downstream mechanism, Akt, MAPK, and NF-κB. Therefore, these results again demonstrate that GSS enhances the immune function of macrophages by inducing the activation of TLR4.

### 3.8. UHPLC-UV-MS/MS Analysis of GSS

**Figure 8:**
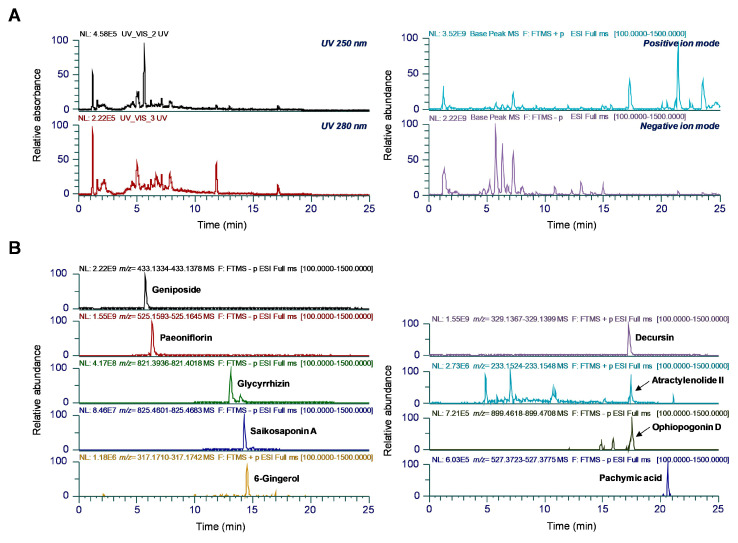
UHPLC-UV-HRMS analysis of nine components in the GSS. (**A**) UV and ion chromatograms of GSS measured at 250 nm and 280 nm wavelengths. (**B**) Extracted ion chromatograms displaying the nine identified components in both positive and negative ionization modes.

To identify phytochemicals in GSS, we conducted a qualitative analysis using UHPLC-UV-MS/MS. The combination of both positive and negative ionization modes in the MS full-scan mode provided additional confidence in the MS spectrum acquisition and molecular mass measurements of each analyte. Figure 8 shows the UV and ion chromatograms of GSS at wavelengths of 250 nm and 280 nm and the extracted ion chromatograms for the precursor ion *m*/*z* values of each analyte. In total, nine major components, namely atractylenolide II from *A. macrocephala*; paeoniflorin from *P. lactiflora* and *P. suffruticosa*; pachymic acid from *P. cocos*; saikosaponin A from *B. falcatum*; decursin from *A*. *gigas*; ophiopogonin D from *L. platyphylla*; glycyrrhizin from *G*. *uralensis*; 6-gingerol from *Z. officinale*; and geniposide from *G. jasminoides*, were identified [17,18,19,20,21]. Most terpenoids and glycosides, saponins, and iridoid glycosides were more ionized in the negative ion mode, whereas other components, such as sesquiterpenes, coumarin and phenolic compounds, were detected in the positive ion mode. Detailed MS data are provided in Table 3. The retention times, *m*/*z* values, and MS2 fragmentation patterns of each analyte were compared with those of the reference standards.

## 4. Discussion

The immune response maintains health by protecting the body from external pathogenic microorganisms, such as bacteria and viruses [1]. If the immune response weakens, the body becomes vulnerable to diseases such as infection, cancer, obesity, diabetes, and tuberculosis, as determined by the immune status [22]. Thus, a decreased immune response is considered a critical clinical problem [23]. In recent years, multiple studies have sought to enhance immune function without side effects by using natural substances [24]. GSS is a widely used herbal formula in Korea, China, and Japan to treat individuals with menopausal symptoms and their associated conditions [11]. GSS also has several biological activities, including antidepression and antianxiety effects [12,13]. However, no reports have described the molecular mechanisms underlying the immune-enhancing activities of GSS. Thus, this study sought to identify the immunostimulatory mechanisms whereby GSS induces the secretion of immunoregulatory mediators in RAW 264.7 macrophages.

The activation of macrophages is an important event in the innate immunity that is implicated in phagocytosis, recognition of foreign substances, production of antibodies, and abnormal cell killing [3]. Activated macrophages promptly excite the production of immune transport mediators, including NO, iNOS, COX-2, cytokines, and ROS [25]. NO is a lipophilic gas that is an important biomolecule of the immune system. The secretion of NO is directly involved in damaging foreign microorganisms and tumor cells [26,27]. NO is synthesized from iNOS, while PGE_2_ is synthesized by COX-2, and both play important roles in immune regulation [28]. In addition, cytokines are essential in regulating the immune response of macrophages [29]. Thus, we analyzed whether GSS treatment induced the production of immunomodulatory mediators in murine macrophage RAW 264.7 cells. Our results indicated that GSS significantly promoted the secretion of NO and effectively increased the expression level of iNOS and COX-2. In addition, GSS treatment induced the secretion of TNF-α, IL-6, IL-1β, MCP-1, and IL-10 and elevated the expression of their mRNA in RAW 264.7 macrophages. ROS expression is a key indicator in determining the function of macrophages and their critical role in immune regulation and defense [30]. Our data showed that GSS strongly enhanced the ROS fluorescence intensity compared with that in nontreated control cells. Furthermore, since these immunoregulators, including NO, iNOS, COX-2, cytokines, and ROS, can activate the phagocytosis of macrophages, we measured the effect of GSS on augmenting phagocytic function. Consequently, the treatment of GSS produced a concentration-dependent increase in the phagocytosis activity of RAW 264.7 macrophages. We also observed that GSS treatment increased the adhesion function and migration activity of RAW 264.7 macrophages. Thus, GSS could improve immune response via the activation of macrophages.

The immunomodulatory role of macrophages is closely associated with the TLRs on their cell surface. TLRs are members of the pattern-recognition receptor family that is implicated in recognizing the PAMPs of external pathogens, and upon activation can stimulate the immune response by producing immunostimulatory mediators [6,7]. In addition, TLRs are implicated in the preparation and coordination of the acquired immune response through regulating the activation of antigen-presenting cells and essential cytokines [31]. Under macrophage activation, TLRs initiate a signaling cascade that activates the Akt, MAPK, and NF-κB signaling pathways [32]. Among them, the MAPK pathway, which includes ERK, p38, and JNK, has a vital role in innate immune response signaling [33]. The phosphorylation of MAPK subsequently promotes the immunoregulatory mediators by activating NF-κB transcription factors [8]. NF-κB is a crucial regulator of several genes closely related to the immune response [34]. The activation of NF-κB causes the phosphorylation and degradation of IκBα, which then frees p65 for translocation into the nucleus to enhance the expression of genes involved in innate and adaptive immunity [35]. Phosphorylation-activated Akt affects downstream regulatory pathways such as that of NF-κB [36]. Moreover, previous studies reported that Akt, MAPK, and NF-κB signaling activation for macrophage stimulation is dependent on TLR4 [37,38]. Therefore, we used Western blotting to clarify whether GSS activates these signaling pathways in RAW 264.7 macrophages. Our results showed that the phosphorylation of MAPK, including ERK, p38, and JNK, was intensely increased by GSS treatment, which also resulted in the phosphorylation and degradation of IκBα and the translocation of p65 into the nucleus. In addition to the activation of these signaling pathways, GSS increased the phosphorylation of Akt. Furthermore, GSS significantly increased TLR 4 expression and similarly increased MyD88 content. Overall, these results suggest that GSS exerts immunostimulatory effects by activating the Akt, MAPK, and NF-κB signaling pathways, mediated by TLR4. Also, the activation of the Akt, MAPK, and NF-κB signaling pathways by GSS was significantly inhibited by co-treatment with TAK-242, a TLR4 inhibitor, which again demonstrates that the immunostimulatory effects by GSS are mediated by TLR4 activation.

We also carried out phytochemical analyses by UHPLC-UV-HRMS to investigate the relationships between the immunostimulatory effects of GSS and its components. Our analysis identified geniposide, paeoniflorin, glycyrrhizin, saikosaponin A, 6-gingerol, decursin, atractylenolide II, ophiopogonin D, and pachymic acid as the main components of GSS. Several of the constituent herbs and identified components of GSS have been shown to be indicated to have an effect on immune responses. In a previous study, *A. gigas* extract was demonstrated to exert immune-enhancing effects by activating the NF-κB/MAPK signaling pathway and restoring natural killer cell activity and splenocyte proliferation in an immunosuppressed mice model [39]. In addition, the polysaccharides of *P. cocos* have been shown to enhance cellular immune responses by promoting CD8+ T cell proliferation and upregulating Th-1-type cytokine production [40]. Furthermore, feeding *G. uralensis* to yellow catfish induced the activation of TLR-NF-κB and the secretion of IL-1β and IL-8, resulting in enhanced immune function to prevent infection [41]. Moreover, geniposide, a major component of *G. jasminoides* fruit and a component of GSS, improved the immune response and enhanced pathogen resistance by regulating the humoral and cellular immunity of the mud crab *Scylla paramamosain* [42]. Therefore, the immune-enhancing effects of GSS reflect the presence of *A. gigas*, *P. cocos*, *G. uralensis*, and geniposide.

## 5. Conclusions

This study revealed that GSS exerted immune-enhancing activities by activating RAW 264.7 macrophages. This was demonstrated by the increased production of immunoregulatory mediators, including NO, iNOS, COX-2, cytokines, and ROS, in macrophages, and the enhancement of phagocytosis ability, adhesion function, and migration activity. In addition, these mechanism studies demonstrated that GSS stimulated TLR4 to activate the Akt, MAPK, and NF-κB signaling pathways, demonstrating that the immune-enhancing effects of GSS are associated with the regulation of these mechanisms. In addition, referring to phytochemical profiling and previous studies, the constituent herbs and ingredients of GSS, including *A. gigas*, *P. cocos*, *G. uralensis*, and geniposide, are closely related to its immunostimulatory effects. Collectively, these results suggest that GSS has potential value as a candidate for enhancing immunity.

## Figures and Tables

**Table 1:** The herbal ingredients and ratio of GSS.

Scientific Name of Herbs	Medicinal Parts	Composition Ratio (%)
*Atractylodes macrocephala*	Rhizome	11.11
*Paeonia lactiflora*	Root	11.11
*Poria cocos*	Sclerotium	11.11
*Bupleurum falcatum*	Root	11.11
*Angelica gigas*	Root	11.11
*Liriope platyphylla*	Tuber	11.11
*Glycyrrhiza uralensis*	Root and Rhizome	5.56
*Mentha arvensis*	Herba	5.56
*Zingiber officinale*	Rhizome (undried)	5.56
*Gardenia jasminoides*	Fruit	8.33
*Paeonia suffruticosa*	Root Bark	8.33

**Table 2:** Primers used for qPCR.

Target Gene	Reference Sequence	Primer Sequence
TNF-α	NM_013693.3	F: 5′-TTCTGTCTACTGAACTTCGGGGTGATCGGTCC-3′
		R: 5′-GTATGAGATAGCAAATCGGCTGACGGTGTGGG-3′
IL-6	NM_031168.2	F: 5′-TCCAGTTGCCTTCTTGGGAC-3′
		R: 5′-GTGTAATTAAGCCTCCGACTTG-3′
IL-1β	NM_008361.4	F: 5′-ATGGCAACTGTTCCTGAACTCAACT-3′
		R: 5′-CAGGACAGGTATAGATTCTTTCCTTT-3′
MCP-1	NM_011333	F: 5′-GCTACAAGAGGATCACCAGCAG-3′
		R: 5′-GTCTGGACCCATTCCTTCTTGG-3′
IL-10	NM_010548	F: 5′-CGGGAAGACAATAACTGCACCC-3′
		R: 5′-CGGTTAGCAGTATGTTGTCCAGC-3′
iNOS	NM_010927.4	F: 5′-GGCAGCCTGTGAGACCTTTG-3′
		R: 5′-GCATTGGAAGTGAAGCGTTTC-3′
COX-2	NM_011198.4	F: 5′-TGAGTACCGCAAACGCTTCTC-3′
		R: 5′-TGGACGAGGTTTTTCCACCAG-3′
β-actin	NM_007393.5	F: 5′-AGAGGGAAATCGTGCGTGAC-3′
		R: 5′-CAATAGTGATGACCTGGCCGT-3′

F, forward; R, reverse.

**Table 3:** Phytochemical profiling of GSS using UHPLC-UV-MS/MS.

No	*t*_R_ (min)	Chemical Formula	Adduct	Estimated (*m*/*z*)	Calculated (*m*/*z*)	Error (ppm)	Identification	Source
1	5.74	C_17_H_24_O_10_	[M + HCO_2_]^−^	433.1352	433.1356	1.0854	Geniposide	*G. jasminoides*
2	6.33	C_23_H_28_O_11_	[M + HCO_2_]^−^	525.1614	525.1619	0.9621	Paeoniflorin	*P. lactiflora*
3	13.08	C_42_H_62_O_16_	[M − H]^−^	821.3965	821.3977	1.4549	Glycyrrhizin	*G. uralensis*
4	14.26	C_42_H_68_O_13_	[M + HCO_2_]^−^	825.4642	825.4652	1.2356	Saikosaponin A	*B. falcatum*
5	14.52	C_17_H_26_O_4_	[M + Na]^+^	317.1723	317.1726	0.7785	6-Gingerol	*Z. officinale*
6	17.26	C_19_H_20_O_5_	[M + H]^+^	329.1384	329.1383	−0.0420	Decursin	*A. gigas*
7	17.44	C_15_H_20_O_2_	[M + H]^+^	233.1536	233.1536	0.1319	Atractylenolide II	*A. macrocephala*
8	17.56	C_44_H_70_O_16_	[M + HCO_2_]^−^	899.4646	899.4663	1.9107	Ophiopogonin D	*L. platyphylla*
9	20.61	C_33_H_52_O_5_	[M − H]^−^	527.3742	527.3749	1.4012	Pachymic acid	*P. cocos*

## Data Availability

The original contributions presented in this study are included in the article, further inquiries can be directed to the corresponding author.

## References

[1] Batatinha H.A., Biondo L.A., Lira F.S., Castell L.M., Rosa-Neto J.C. (2019). Nutrients, immune system, and exercise: Where will it take us?. Nutrition.

[2] Ren Z., He C., Fan Y., Si H., Wang Y., Shi Z., Zhao X., Zheng Y., Liu Q., Zhang H. (2014). Immune-enhancing activity of polysaccharides from Cyrtomium macrophyllum. Int. J. Biol. Macromol..

[3] Gordon S., Martinez F.O. (2010). Alternative activation of macrophages: Mechanism and functions. Immunity.

[4] Lee S.M., Kim B.N., Kim Y.H., Min J. (2023). Identification of TLR2/4-mediated phagocytosis and immune response activation pathways by vacuoles isolated from Saccharomyces cerevisiae. J. Cell. Biochem..

[5] Unanue E.R. (1984). Antigen-presenting function of the macrophage. Annu. Rev. Immunol..

[6] Fitzgerald K.A., Kagan J.C. (2020). Toll-like receptors and the control of immunity. Cell.

[7] Xu D., Liu H., Komai-Koma M. (2004). Direct and indirect role of Toll-like receptors in T cell mediated immunity. Cell. Mol. Immunol..

[8] Wang N., Liang H., Zen K. (2014). Molecular mechanisms that influence the macrophage M1–M2 polarization balance. Front. Immunol..

[9] Um Y., Eo H.J., Kim H.J., Kim K., Jeon K.S., Jeong J.B. (2020). Wild simulated ginseng activates mouse macrophage, RAW264. 7 cells through TRL2/4-dependent activation of MAPK, NF-κB and PI3K/AKT pathways. J. Ethnopharmacol..

[10] Heo J. (1994). Donguibogam.

[11] Scheid V., Ward T., Cha W.S., Watanabe K., Liao X. (2010). The treatment of menopausal symptoms by traditional East Asian medicines: Review and perspectives. Maturitas.

[12] Kim J.Y., Kwak D.H., Ju E.J., Kim S.M., Lee D.H., Keum K.S., Lee S.U., Jung K.Y., Seo B.B., Choo Y.K. (2004). Effects of Gamisoyosan on in vitro fertilization and ovulation of stressed mice by electric shock. Arch. Pharm. Res..

[13] Yamada K., Kanba S. (2007). Effectiveness of kamishoyosan for premenstrual dysphoric disorder: Open-labeled pilot study. Psychiatry Clin. Neurosci..

[14] Jin S.W., Lee G.H., Jang M.J., Hong G.E., Kim J.Y., Park G.D., Jin H., Kim H.S., Choi J.H., Choi C.Y. (2020). Immunomodulatory activity of Lactococcus lactis GCWB1176 in cyclophosphamide-induced immunosuppression model. Microorganisms.

[15] Li Y., Yu P., Fu W., Cai L., Yu Y., Feng Z., Wang Y., Zhang F., Yu X., Xu H. (2021). Ginseng–Astragalus–oxymatrine injection ameliorates cyclophosphamide-induced immunosuppression in mice and enhances the immune activity of RAW264.7 cells. J. Ethnopharmacol..

[16] Pak M.E., Park Y.J., Yang H.J., Hwang Y.H., Li W., Go Y. (2022). Samhwangsasim-tang attenuates neuronal apoptosis and cognitive decline through BDNF-mediated activation of tyrosin kinase B and p75-neurotrophin receptors. Phytomedicine.

[17] Jung M.Y., Seo C.S., Baek S.E., Lee J., Shin M.S., Kang K.S., Lee S., Yoo J.E. (2019). Analysis and Identification of Active Compounds from Gami-Soyosan Toxic to MCF-7 Human Breast Adenocarcinoma Cells. Biomolecules.

[18] Jin S.E., Kim O.S., Yoo S.R., Seo C.S., Kim Y., Shin H.K., Jeong S.J. (2016). Anti-inflammatory effect and action mechanisms of traditional herbal formula *Gamisoyo-san* in RAW 264.7 macrophages. BMC Complement. Altern. Med..

[19] Lu C.M., Hou M.L., Lin L.C., Tsai T.H. (2013). Chemical and Physical Methods to Analyze a Multicomponent Traditional Chinese Herbal Prescription Using LC-MS/MS, Electron Microscope, and Congo Red Staining. Evid. Based Complement. Alternat. Med..

[20] Park S.H., Lee H.J., Ryu J., Son K.H., Kwon S.Y., Lee S.K., Kim Y.S., Hong J.H., Seok J.H., Lee C.J. (2014). Effects of ophiopogonin D and spicatoside A derived from Liriope Tuber on secretion and production of mucin from airway epithelial cells. Phytomedicine.

[21] Ling Y., Chen M., Wang K., Sun Z., Li Z., Wu B., Huang C. (2012). Systematic screening and characterization of the major bioactive components of *Poria cocos* and their metabolites in rats by LC-ESI-MS^n^. Biomed. Chromatogr..

[22] Kumar S., Sharma G., Sidiq T., Khajuria A., Jain M., Bhagwat D., Dhar K. (2014). Immunomodulatory potential of a bioactive fraction from the leaves of Phyllostachys bambusoides (Bamboo) in BALB/C mice. EXCLI J..

[23] Jeong D.Y., Yang H.J., Jeong S.J., Kim M.G., Yun C.Y., Lee H.Y., Lee Y.H., Shin D.Y., Lee H.S., Park Y.M. (2019). Immunostimulatory effects of blueberry yeast fermented powder against cyclophosphamide-induced immunosuppressed model. J. Physiol. Pathol. Korean Med..

[24] Promphet P., Bunarsa S., Sutheerawattananonda M., Kunthalert D. (2014). Immune enhancement activities of silk lutein extract from Bombyx mori cocoons. Biol. Res..

[25] Mosser D.M., Edwards J.P. (2008). Exploring the full spectrum of macrophage activation. Nat. Rev. Immunol..

[26] Olefsky J.M., Glass C.K. (2010). Macrophages, inflammation, and insulin resistance. Annu. Rev. Physiol..

[27] Nathan C., Shiloh M.U. (2000). Reactive oxygen and nitrogen intermediates in the relationship between mammalian hosts and microbial pathogens. Proc. Natl. Acad. Sci. USA.

[28] Guzik T., Korbut R., Adamek-Guzik T. (2003). Nitric oxide and superoxide in inflammation. J. Physiol. Pharmacol..

[29] Sudeep H.V., Gouthamchandra K., Ramanaiah I., Raj A., Naveen P., Shyamprasad K. (2023). A standardized extract of Echinacea purpurea containing higher chicoric acid content enhances immune function in murine macrophages and cyclophosphamide-induced immunosuppression mice. Pharm. Biol..

[30] Martinvalet D., Walch M. (2022). The role of reactive oxygen species in protective immunity. Front. Immunol..

[31] Duan T., Du Y., Xing C., Wang H.Y., Wang R.F. (2022). Toll-like receptor signaling and its role in cell-mediated immunity. Front. Immunol..

[32] Qu Z., Chen Y., Luo Z.H., Shen X.L., Hu Y.J. (2020). 7-methoxyflavanone alleviates neuroinflammation in lipopolysaccharide-stimulated microglial cells by inhibiting TLR4/MyD88/MAPK signalling and activating the Nrf2/NQO-1 pathway. J. Pharm. Pharmacol..

[33] Dong C., Davis R.J., Flavell R.A. (2002). MAP kinases in the immune response. Annu. Rev. Immunol..

[34] Chapman N.R., Perkins N.D. (2000). Inhibition of the RelA (p65) NF-κB subunit by Egr-1. J. Biol. Chem..

[35] Zhang Y., Zhang A., Zhao Y., Feng X., Sheng Y., Zhang H., Weng Q., Xu M. (2019). Expressions of TLR4, MyD88, IRAK4 and NF-ÎB in the oviduct of Chinese brown frog (*Rana dybowskii*). Eur. J. Histochem..

[36] Dan H.C., Cooper M.J., Cogswell P.C., Duncan J.A., Ting J.P.Y., Baldwin A.S. (2008). Akt-dependent regulation of NF-κB is controlled by mTOR and Raptor in association with IKK. Genes Dev..

[37] Shen T., Wang G., You L., Zhang L., Ren H., Hu W., Qiang Q., Wang X., Ji L., Gu Z. (2017). Polysaccharide from wheat bran induces cytokine expression via the toll-like receptor 4-mediated p38 MAPK signaling pathway and prevents cyclophosphamide-induced immunosuppression in mice. Food Nutr. Res..

[38] Yang Y., Xing R., Liu S., Qin Y., Li K., Yu H., Li P. (2018). Immunostimulatory effects of sulfated chitosans on RAW 264.7 mouse macrophages via the activation of PI3 K/Akt signaling pathway. Int. J. Biol. Macromol..

[39] Jeong J., Lim M.K., Han E.H., Lee S.H., Lee S. (2023). Immune-enhancement effects of *Angelica gigas* Nakai extracts via MAPK/NF-ƙB signaling pathways in cyclophosphamide-induced immunosuppressed mice. Food Sci. Biotechnol..

[40] Dong X., Li B., Yu B., Chen T., Hu Q., Peng B., Sheng W. (2021). Poria cocos polysaccharide induced Th1-type immune responses to ovalbumin in mice. PLoS ONE.

[41] Wang Q., Shen J., Yan Z., Xiang X., Mu R., Zhu P., Yao Y., Zhu F., Chen K., Chi S. (2020). Dietary Glycyrrhiza uralensis extracts supplementation elevated growth performance, immune responses and disease resistance against *Flavobacterium columnare* in yellow catfish (*Pelteobagrus fulvidraco*). Fish Shellfish Immunol..

[42] Gong J., Jin Q., Zhu F. (2024). Effects of geniposide on innate immunity and antiviral activity of Scyllaparamamosain. Fish Shellfish Immunol..

